# Clinical value of digital tomographic fusion imaging in the diagnosis of avascular necrosis of the femoral head in adults

**DOI:** 10.1007/s11845-020-02451-9

**Published:** 2021-01-06

**Authors:** Jiangang Zhang, Zhuhai Wang, Ge Hong

**Affiliations:** Radiology Department, Third Hospital of Hebei Medical University, Shijiazhuang, China

**Keywords:** Avascular necrosis of the femoral head, Digital tomography fusion imaging

## Abstract

**Background:**

To explore the clinical significance of digital tomographic fusion imaging in the diagnosis of avascular disease of the femoral head in adults.

**Methods:**

Eighty-two adult patients with avascular necrosis of the femoral head confirmed by MRI in the department of orthopedics of our hospital were studied retrospectively. The related signs of adult avascular necrosis of the femoral head were diagnosed by digital tomographic fusion imaging, and the detection rates of digital X-ray (DR) and digital tomosynthesis (DTS) were compared to clarify the clinical value of digital tomographic fusion imaging in the diagnosis of adult avascular necrosis of the femoral head.

**Results:**

DTS detected DR and 78 cases identified 55 cases. Taking the results of CT/MRI as the gold standard, the sensitivity, specificity, positive predictive value, and negative predictive value of DR and DTS in the diagnosis of ANFH were calculated. There was a significant difference in the detection rate between the two methods (*P* < 0.05).

**Conclusion:**

The digital tomographic fusion imaging technique has the advantages of high detection rate and excellent image quality, is economical, and is worth popularizing. For those with negative X-rays, DTS diagnosis and CT or/and MRI can avoid unnecessary CT and MRI examinations, which is helpful to reduce the waste of medical resources.

Avascular necrosis of the femoral head (avascular necrosis of the femoral head, ANFH) is a typical bone and joint disease in China, mostly in young adults, with a high disability rate, which seriously affects the work and study of patients, reduces their quality of life, and does great harm to the physical and mental health of our people and the cause of socialist construction [1]. Digital X-ray is generally the first choice for examination, but DR can only display compound images. Due to the overlap of anatomical structures, it is challenging to show fractures, dislocations, and healthy anatomical tissues. The early standard plain film of ANFH may be misconfirmed and delay the appropriate referral time. If the plain film is negative and the patient continues to complain about hip discomfort, the doctor may diagnose non-specific hip pain and send the patient to physiotherapy [2], but because bone destruction occurs within 2 years after onset, it is essential for early diagnosis and early treatment of ANFH.

At present, CT and MRI are the main imaging methods for the diagnosis of early avascular necrosis of the femoral head, but these two examinations are much more expensive than ordinary radiological examination, and each has different taboo signs. The taboo sign of MRI limits a considerable number of patients with avascular necrosis of the femoral head. Digital tomographic fusion (digital tomosynthesis, DTS) has greater advantages than digital X-ray in image display and diagnostic accuracy of some deep and complex parts [3], especially in breast and chest imaging. Orthopedics such as the spine [4], foot [5], hand [6], knee joint [7], and hip joint [8] have been studied, showing that DTS is of great value in the skeletal system. Alice S. Ha et al. proposed that DTS and CT can overcome overlapping bones by plane-by-plane function, thus avoiding the need for additional special views [9]. Specifically, for the digital tomographic fusion imaging of the hip joint, a series (60 images) in the range of 40 (± 20) angles takes only about 10 s, the image is a continuous linear motion, and the radiation dose is much less than the CT scan of a specific area [10]. GOMIT et al. have studied that through different DT algorithms, the interference of metal artifacts to the image can be reduced, and the minimum range of metal artifacts can be controlled in 0.54–0.33 mSv [11]. Oliver J Gurney-Champion et al. proposed that DTS can be accurately positioned without rotation [12]. The economic cost of digital tomographic fusion is much lower than that of MRI. It can provide high-quality digital tomographic images without CT and MRI and has great potential in the diagnosis of avascular necrosis of the femoral head in adults.

Here, we examine the related signs of adult ANFH diagnosed by DTS and the detection rates of DR and DTS to clarify the clinical value of digital tomographic fusion imaging in the diagnosis of adult avascular necrosis of the femoral head.

## Data and methods

### General information

This retrospective study included 178 patients with hip pain or claudication from April 2017 to December 2019 in our hospital. All patients were examined by DR, DTS, CT, and MRI, as directed by orthopedic doctors. Eighty-two cases were diagnosed as adult avascular necrosis of the femoral head. Most of the selected cases had a history of hip trauma, large alcohol intake, and hormone use, and the clinical manifestations were hip pain or claudication. Physical examination: the range of motion of the hip joint was limited in varying degrees; the “4” sign was positive and so on.

### Examination instrument

1. GEDiscoveryXR650 digital radiography system
2. AS Edge64 layer spiral CT
3. Symphony, Avanto, VerioMRI scanner

### Methods

All the patients in this study were examined by DR, CT, or MRI and digital tomographic fusion imaging, and the interval of all imaging examinations was less than 1 week.

Digital tomographic fusion imaging technology (DTS) and digital radiography (digital radiography, DR) protect non-irradiated areas during exposure. The position of the patient was standard pelvic anterior and posterior, the patient was lying on his back, the internal rotation of both lower limbs was 15 °, and the source-image distance was 120 cm. The X-ray incident direction was aimed at the midpoint between the superior edge of the pubic symphysis and the line of the bilateral anterior superior iliac spine. The photography condition is 95 kV, 300 mA, 35 cm × 41 cm scanning field (FOV), automatic exposure control system (AEC) automatic exposure, the scanning parameters of DTS examination are 95KV, 500 mA, 1.01mAs, 2.0 ms, 35 cm × 41cmFOV, seven times dose ratio, reconstruction start height 50 mm, end height 150 mm, layer spacing 5 mm, 1 mm, acquisition factor is 1, using fixed (FIXED) exposure mode, no ionization chamber, image matrix 1024 × 1024.

#### CT scan

Parameters: pitch 0.9, visual field 300 mm, voltage 120 kV, current using automatic millisecond

#### MRI scan

The patients were taken a prone position and scanned continuously from 3 cm above acetabulum to subtrochanteric of the femur. Slice thickness 5 mm was scanned continuously. T1WI, T2WI, and STIR coronal scan of spin-echo SE sequence and axial T1WI scan were selected. The interval of all imaging examinations is no more than 1 week.

#### Imaging analysis and staging

Two radiologists analyzed the imaging data of the patients, and the consensus on the diagnosis and staging of ONFH was obtained. If there is disagreement, please ask another chief radiologist to confirm it. Using the concept of early diagnosis of avascular necrosis of the femoral head defined by the scholar Li Zirong, the early avascular necrosis of the femoral head was defined as ARCO I~ARCOII.

#### 1Statistical analysis

Using SPSS26.0 statistical analysis software, taking the results of MRI as the gold standard, the sensitivity, specificity, positive predictive value, and negative predictive value of DR, digital tomographic fusion imaging and CT in the diagnosis of ANFH were calculated. Among them, a *t* test was used to compare the measurement data, the chi-square test was used to compare the counting data, and the difference was statistically significant (*P* < 0.05).

## Result

One hundred seventy-eight patients were followed up, including 107 males and 71 females, aged 48.6 years (range: 21–73).

**Fig. 1:**
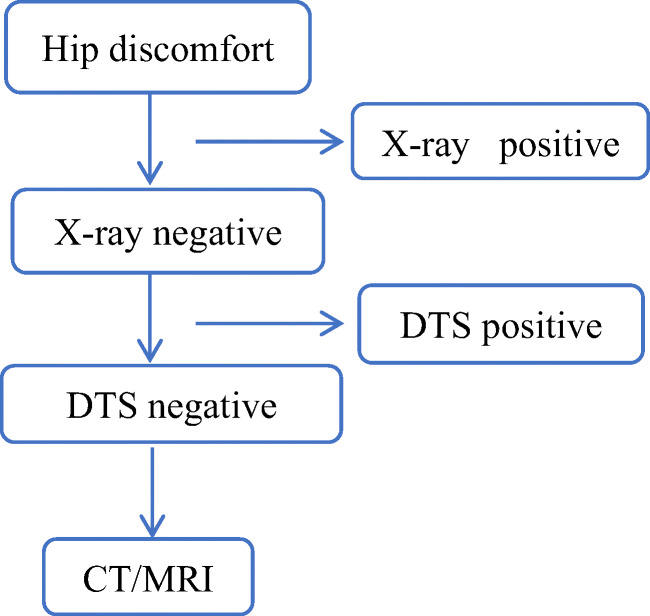
Flowchart of patient examination

**Fig. 2:**
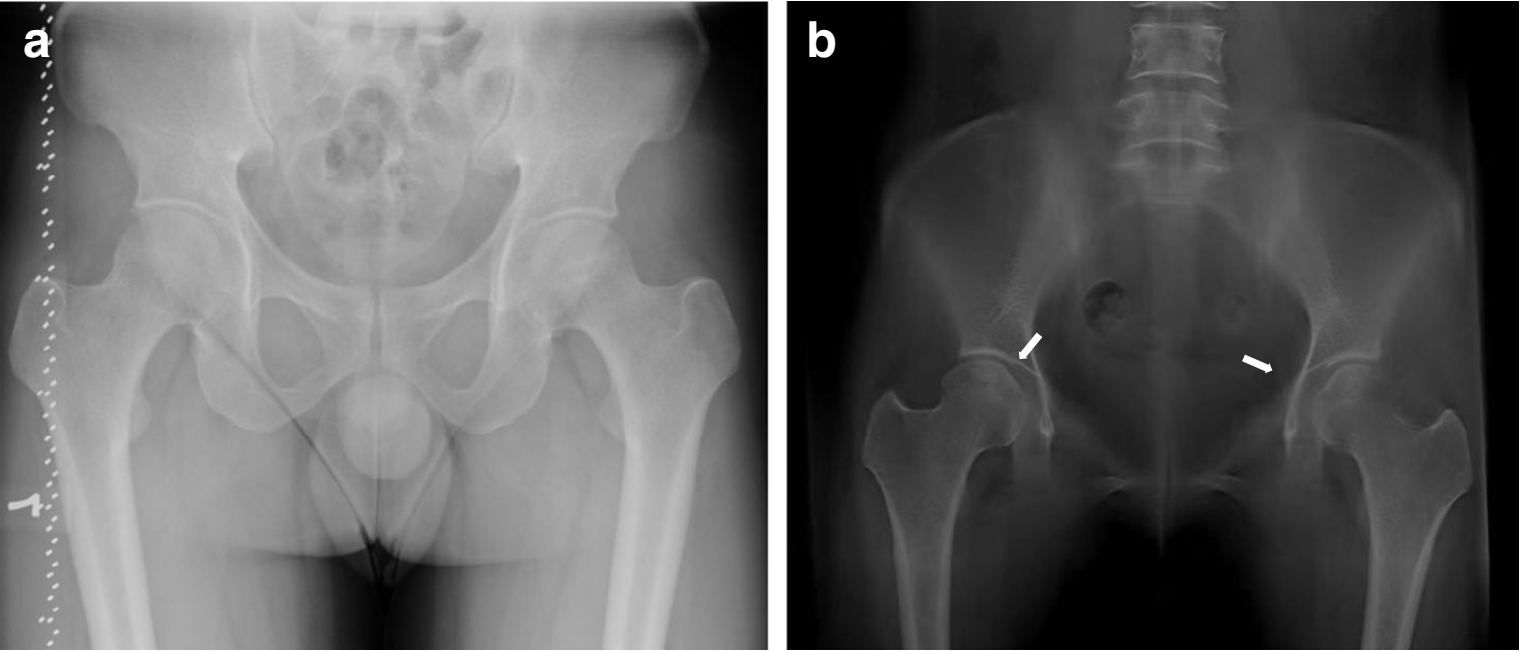
**a** DR images show suspected osteonecrosis of the femoral head. **b** DTS images show a definite diagnosis of osteonecrosis of the femoral head

**Fig. 3:**
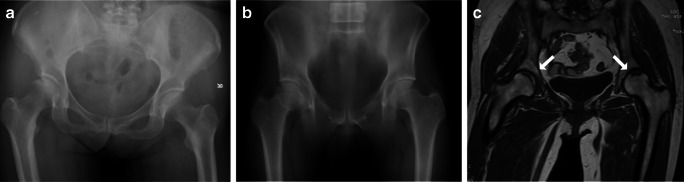
**a** DR shows no apparent abnormality. **b** DTS shows (**c**) CT/MR diagnosis of osteonecrosis of the femoral head

The main results were as follows: (1) A total of 178 patients with hip discomfort were examined by DR, DTS, CT, and/or MRI. Two radiologists analyzed the imaging data of the patients. The experimental results can prove that when the clinic uses DTS first, it can reduce the unnecessary CT/MRI examination, shorten the examination time, reduce the medical cost, avoid unnecessary radiation exposure, and avoid the waste of medical resources. DTS shows its unique advantages in the treatment of osteonecrosis of the femoral head which cannot be detected by plain X-ray film. This experiment confirms this point of view. The examination procedure of patients and the comparison of DR and DTS images are shown in Figs. 1, 2, and 3.

(2) The patients suspected of osteonecrosis of the femoral head were examined by X-ray and DTS equipment. CT/MR was used as the reference standard, and two readers compared the results. The results showed that 82 lesions were diagnosed in 178 subjects. The sensitivity, specificity, positive predictive value, negative predictive value, accuracy, and confidence of the two imaging methods in the diagnosis of avascular necrosis of the femoral head were compared and analyzed by two diagnostic physicians. The results of X-ray plain film and DTS imaging are shown in Table 1.

**Table 1:** Comparison of X-ray and DTS results

Diagnostics	Sensitivity		Specificity		Positive predictive value	
	X-ray	DTS	X-ray	DTS	X-ray	DTS
Viewer 1	60.98% (50/82)	91.46% (75/82)	36.46% (35/96)	90.63% (87/96)	45.06% (50/111)	89.29% (75/84)
Viewer 2	67.07% (55/82)	95.12% (78/82)	37.50% (36/96)	90.63% (87/96)	47.83% (55/115)	89.66% (78/87)
Diagnostics	Negative predictive value		Accuracy		Diagnostic confidence (95%CI)	
	X-ray	DTS	X-ray	DTS	X-ray	DTS
Viewer 1	52.24% (35/67)	92.55% (87/94)	47.75% (85/178)	91.01% (162/178)	0.799 (0.733~0.865)	0.948 (0.914~0.982)
Viewer 2	57.14% (36/63)	95.60% (87/91)	51.12% (91/178)	92.70% (165/178)	0.831 (0.772~0.891)	0.973 (0.949~0.997)

## Conclusions

DR is a routine and preferred examination for avascular necrosis of the femoral head because of its rapid and straightforward operation, low price, and low radiation dose. DR can display the full picture of the hip joint and the femoral head, observe the changes of the joint space, and determine whether there is bone destruction, but because the DR images of some patients are not typical, it is easy to cause missed diagnosis and misdiagnosis. This study found that in the diagnosis of ANFH, the sensitivity of DR is less than 70%, with poor specificity and accuracy, indicating that DR still has limitations in the diagnosis of ANFH. While the detection rate, sensitivity, and specificity of DTS are more than 90%, which is much higher than that of DR, indicating the clinic value of DTS when diagnosing ANFH.

## Discussion

ANFH is a common bone and joint disease in China, with a high disability rate. Bone destruction will occur 2 years after the onset of the disease. If physical therapy is misjudged as non-specific pain and delay in the treatment of the disease itself, it will bring valuable losses to young and middle-aged adults, so early intervention is significant for the early diagnosis of the disease. DTS can be used to detect very delicate bone structures, which is not affected by the overlap of surrounding tissues and can be played continuously. It can provide researchers and doctors with the relationship between the research site and the surrounding tissues and provide more information for clinical and scientific research. Therefore, DTS has a good prospect in the diagnosis of ANFH.

In this retrospective study, it is found that the detection rate, sensitivity, and specificity of DTS are more than 90%, which is much higher than that of DR, Therefore, for patients with hip discomfort, DTS can be performed directly, which can reduce unnecessary MRI and CT tests for some patients, avoid waste of medical resources, reduce radiation, and increase patient care.

The spatial resolution of DTS is higher than that of DR, and the detection rate, sensitivity, and specificity are higher than that of DR. The economic cost of radiation dose less than CT is only slightly higher than that of DR, much less than that of CT and MRI, the imaging time is short, and the operation is simple and easy. Considering comprehensively, DTS has essential value in the diagnosis of avascular necrosis of the femoral head and is worth popularizing and applying.

## Abbreviations

DR: Digital X-ray
DTS: Digital tomosynthesis
ANFH: Avascular necrosis of the femoral head

## References

[1] da Silva RS, Marins LF, Almeida DV (2019). A comparison of classifiers for predicting the class color of fluorescent proteins. Comput Biol Chem.

[2] Pijnenburg L, Felten R, Javier RM (2020). A review of avascular necrosis, of the hip and beyond. Rev Med Interne.

[3] Debbi EM, Rajaee SS, Mayeda BF, Penenberg BL (2019). Determining and achieving target limb length and offset in total hip arthroplasty using intraoperative digital radiography. J Arthroplast.

[4] Berggren K, Cederstrom B, Lundqvist M, Fredenberg E (2018). Technical note: comparison of first- and second-generation photon-counting slit-scanning tomosynthesis systems. Med Phys.

[5] Koo BS, Song Y, Lee S (2017). Prevalence and distribution of sesamoid bones and accessory ossicles of the foot as determined by digital tomosynthesis. Clin Anat.

[6] Koo BS, Song Y, Sung YK (2017). Prevalence and distribution of sesamoid bones in the hand determined using digital tomosynthesis. Clin Anat.

[7] Liu JN, Shields TG, Gowd AK, Amin NH (2019). Surgical treatment of insufficiency fractures of the knee. Arthrosc Tech.

[8] Gillet R, Teixeira P, Bonarelli C (2019). Comparison of radiographs, tomosynthesis and ct with metal artifact reduction for the detection of hip prosthetic loosening. Eur Radiol.

[9] Ha AS, Cunningham SX, Leung AS (2019). Weightbearing digital tomosynthesis of foot and ankle arthritis: comparison with radiography and simulated weightbearing ct in a prospective study. AJR Am J Roentgenol.

[10] Al-Mokhtar N, Shah J, Marson B (2015). Initial clinical experience of the use of digital tomosynthesis in the assessment of suspected fracture neck of femur in the elderly. Eur J Orthop Surg Traumatol.

[11] Gomi T, Sakai R, Goto M (2018). Development of a novel algorithm for metal artifact reduction in digital tomosynthesis using projection-based dual-energy material decomposition for arthroplasty: a phantom study. Phys Med.

[12] Oravec D, Flynn MJ, Zauel R (2019). Digital tomosynthesis based digital volume correlation: a clinically viable noninvasive method for direct measurement of intravertebral displacements using images of the human spine under physiological load. Med Phys.

